# Exploiting differences in the energy budget among C_4_ subtypes to improve crop productivity

**DOI:** 10.1111/nph.17011

**Published:** 2020-11-20

**Authors:** Xinyou Yin, Paul C. Struik

**Affiliations:** ^1^ Centre for Crop Systems Analysis Department of Plant Sciences Wageningen University & Research PO Box 430 Wageningen 6700 AK the Netherlands

**Keywords:** C_4_ photosynthesis, crop improvement, energy budget, photosynthesis ideotype, quantum yield

## Abstract

C_4_ crops of agricultural importance all belong to the NADP‐malic enzyme (ME) subtype, and this subtype has been the template for C_4_ introductions into C_3_ crops, like rice, to improve their productivity. However, the ATP cost for the C_4_ cycle in both NADP‐ME and NAD‐ME subtypes accounts for > 40% of the total ATP requirement for CO_2_ assimilation. These high ATP costs, and the associated need for intense cyclic electron transport and low intrinsic quantum yield $\Phi_{{\text{CO}}_{2}}$, are major constraints in realizing strong improvements of canopy photosynthesis and crop productivity. Based on mathematical modelling, we propose a C_4_ ideotype that utilizes low chloroplastic ATP requirements present in the nondomesticated phospho*enol*pyruvate carboxykinase (PEP‐CK) subtype. The ideotype is a mixed form of NAD(P)‐ME and PEP‐CK types, requires no cyclic electron transport under low irradiances, and its theoretical $\Phi_{{\text{CO}}_{2}}$ is *c*. 25% higher than that of a C_4_ crop type. Its cell‐type‐specific ATP and NADPH requirements can be fulfilled by local energy production. The ideotype is projected to have *c*. 10% yield advantage over NADP‐ME‐type crops and > 50% advantage over C_3_ counterparts. The ideotype provides a unique (theoretical) case where $\Phi_{{\text{CO}}_{2}}$ could be improved, thereby paving a new avenue for improving photosynthesis in both C_3_ and C_4_ crops.

## Introduction

Increasing yields of major food crops, like wheat and rice, to cope with increasing populations is a major challenge in agriculture, especially in the face of an accelerating climate change (Fischer *et al*., 2014). Crop yield improvement by the Green Revolution in the 1960s has resulted almost exclusively from the increased harvest index by introducing (semi)dwarfing genes, which allowed more fertilizer input without the risk of having more lodging (Fischer *et al*., 2014). Afterwards, crop yield potential has been improved progressively; for example, by optimizing morphological characteristics and exploiting heterosis (Yuan, 2017). Since the benefit from approaches such as improving harvest index may have reached a maximum value, further improvement of crop productivity has to come from significantly improved crop photosynthesis competence, either by increasing leaf photosynthesis efficiency or by improving light interception, or by optimizing the profile of photosynthetic resources in the crop canopy (Zhu *et al*., 2010).

Whereas conventional breeding may have selected photosynthesis‐related traits that likely contributed partly to recent yield progress (Fischer *et al*., 2014), the natural variation in leaf photosynthesis above the check variety within a species is generally small (Gu *et al*., 2014). Therefore, several research consortia have aimed to introduce C_4_ photosynthesis (as in maize) into C_3_ crops (like rice) via genetic engineering (e.g. von Caemmerer *et al*., 2012; Schuler *et al*., 2016), and significant progress has already been made – see the recent reviews of Weber & Bar‐Even (2019) and Ermakova *et al*. (2020). The rationale for these endeavours is the CO_2_‐concentrating mechanism (CCM) in C_4_ leaves that largely suppresses photorespiration, thereby resulting in higher light‐saturated maximum leaf photosynthetic capacity *A*_max_ (see Supporting Information Table S1 for symbol definitions) in C_4_ than C_3_ leaves. It was hoped that rice productivity could be increased by 50% when initially conceiving the C_4_ rice project (Mitchell & Sheehy, 2006). However, more recent analysis, using a crop model based on more detailed physiological parameters, showed that potential productivity of rice when grown under the current climatic condition could be increased by only 33–42% by introducing the complete maize‐like C_4_ mechanism, and the advantage was even lower under future high‐CO_2_ atmospheric conditions (Yin & Struik, 2017). This less‐than‐expected advantage, as also predicted by Bellasio & Farquhar (2019), has been explained by little gain of quantum yield of photosynthetic CO_2_ assimilation $\Phi_{{\text{CO}}_{2}}$ in maize‐C_4_ relative to C_3_ plants, because the photosynthetic rate of lower leaves in a full canopy is largely determined by $\Phi_{{\text{CO}}_{2}}$ rather than by *A*_max_. Many studies (e.g. Day & Chalabi, 1988; Gu *et al*., 2014) showed that full‐canopy photosynthesis generally depends more on $\Phi_{{\text{CO}}_{2}}$ than on *A*_max_.

The small advantage of $\Phi_{{\text{CO}}_{2}}$ in maize relative to rice leaves is because the operation of CCM in maize leaves requires ATP and the production of ATP is limited under low light conditions prevalent in lower leaves of a canopy. The Calvin cycle for assimilating 1 mol CO_2_ requires 2 mol NADPH and 3 mol ATP, whereas the C_4_ cycle in maize additionally requires 2 mol ATP because of the regeneration of phospho*enol*pyruvate (PEP) by pyruvate, phosphate dikinase (PPDK) in the C_4_ cycle (Hatch, 1987). This means that the C_4_ cycle accounts for *c*. 40% of the total ATP consumption (von Caemmerer & Furbank, 1999). As the generally believed stoichiometry of linear electron transport (LET) in chloroplasts gives an ATP : NADPH ratio of 1.5 (Allen, 2003), the ratio exactly matching the required ratio by the Calvin cycle, the ATP required for the C_4_ cycle has to come from the cyclic electron transport (CET; Yin & Struik, 2012). In the C_4_ cycle, bicarbonate ions, converted from CO_2_, are fixed in mesophyll (M) cells by PEP carboxylase (PEPc) to produce oxaloacetate (OAA); OAA is quickly reduced to malate or converted to aspartate. These C_4_ acids move to bundle‐sheath (BS) cells, where they are decarboxylated to deliver CO_2_ to Rubisco to start the Calvin cycle. There are different enzymes for decarboxylating C_4_ acids in BS compartments, and C_4_ species have been classified traditionally into three subtypes according to the decarboxylating enzyme: NADP‐malic enzyme (ME) in chloroplasts, NAD‐ME in mitochondria, and PEP‐carboxykinase (CK) in the cytosol (Hatch, 1987; Fig. S1).

In NADP‐ME and NAD‐ME subtypes, decarboxylating malate into CO_2_ produces pyruvate. Pyruvate moves directly (in the NADP‐ME subtype) or via aminotransferase (in the NAD‐ME subtype) to M cells, where pyruvate reacts to regenerate PEP by PPDK (Fig. S1a,b). Based on the NADPH and ATP requirement and generally estimated leakiness *ϕ*, the fraction for the CO_2_ leakage from BS to M cells, Yin & Struik (2018) theoretically calculated that $\Phi_{{\text{CO}}_{2}}$ of the two ME subtypes under the atmospheric condition was *c*. 0.064 mol mol^−1^ (Table 1), comparable to the measured $\Phi_{{\text{CO}}_{2}}$ (Ehleringer & Pearcy, 1983). These values are not much higher than $\Phi_{{\text{CO}}_{2}}$ of C_3_ species (Ehleringer & Pearcy, 1983) if adjusted for reference temperature 25°C under the current atmospheric CO_2_ conditions. This indicates that, at low irradiances, the gain from CCM in minimizing photorespiration is largely cancelled out by the extra ATP requirement for CCM. C_4_ crops of agricultural importance for food and energy (maize, sorghum, millet, sugarcane, and *Miscanthus × giganteus*) all belong to the NADP‐ME subtype (Hatch, 1987; Sage, 2016), and the subtype has been used as the template for developing C_4_ rice (Karki *et al*., 2013).

**Table 1:** NADPH and ATP requirement from chloroplastic electron transport (mol mol^−1^ CO_2_) for the Calvin cycle (before ‘+’ sign) and for the C_4_ cycle (after ‘+’ sign), and theoretical quantum yield of CO_2_ assimilation ($\Phi_{{\text{CO}}_{2}}$, mol mol^−1^) for various (proposed) types of C_4_ photosynthesis, using $\Phi_{{\text{CO}}_{2}}$ of C_3_ photosynthesis under the nonphotorespiratory (NPR) condition as the reference.

			Minimum energy required from chloroplast^a^		*f*_CET_	$\Phi_{{\text{CO}}_{2}}$	*x*
			NADPH	ATP			
C_3_ (NPR)			2	3	0	0.100	na
Classical C_4_ subtypes	NADP‐ME		2	3 + 2	0.5	0.064	0.41
	NAD‐ME		2	3 + 2	0.5	0.064	0.41
	PEP‐CK	*n* = 3	2 + 0.25	3 + 0.50	0.05	0.081	0.15
		*n* = 2.5	2 + 0.286	3 + 0.572	0.06	0.080	0.17
NH_2_‐balanced PEP‐CK type^b^		*n* = 3	2 + 0.5	3 + 0.0	0	0.075	0.00
		*n* = 2.5	2 + 0.5	3 + 0.25	0	0.075	0.09
C_4_ ideotype^b^		*n* = 3	2 + 0.36	3 + 0.55	0	0.079	0.16
		*n* = 2.5	2 + 0.4	3 + 0.6	0	0.078	0.17

*x*, the proportion of the chloroplastic ATP that is used to support the C_4_ or CO_2_‐concentrating mechanism cycle; na, not applicable.

*n*, stoichiometric coefficient for the number of ATP generated per NADH oxidation; *f*_CET_, $\Phi_{{\text{CO}}_{2}}$, and *x* in the last three columns were estimated assuming that all C_4_ types had the same leakiness (0.16; Yin & Struik, 2012) and the same proportions for alternative energy sinks (see Supporting Information Table S2), to allow objective comparison of their intrinsic quantum yield.

^a^ The word ‘Minimum’ is used because data in these columns do not include energy loss due to leakiness and energy requirements by alternative energy sinks.

^b^ NADPH and ATP requirements for these types are derived from discussion in the text; *f*_CET_, fraction for cyclic electron transport.

In the PEP‐CK subtype, a portion of OAA is reduced to malate, using NADPH from M chloroplasts, and its remaining portion moves, via aspartate, to the BS cytosol. The OAA in the BS cytosol is directly decarboxylated by PEP‐CK while NAD‐ME simultaneously decarboxylates malate in the BS mitochondria (Hatch, 1987; Fig. S1c). Although this PEP‐CK subtype requires additional NADPH to operate, it is the most efficient C_4_ type in terms of ATP requirement from the chloroplast electron transport chain. First, only 1 mol ATP is required per mole of OAA decarboxylated by PEP‐CK into CO_2_ and PEP (Hatch, 1987; Kanai & Edwards, 1999), and the PEP returns directly to the M cytosol, thereby minimizing the use of PPDK that would need 2 mol ATP to regenerate 1 mol PEP. Second, the ATP required by PEP‐CK can be exclusively produced by NADH oxidation in the respiratory chain of BS mitochondria associated with malate decarboxylation (Hatch, 1987; Burnell & Hatch, 1988). As the stoichiometry of ATP production per oxidation of NADH in mitochondria (the ATP : NADH ratio *n*) is believed to be either 3 (von Caemmerer & Furbank, 1999) or 2.5 (Kanai & Edwards, 1999) – much higher than the generally believed ATP : NADPH ratio of 1.5 for the LET in chloroplasts – the cost for additional NADPH in this subtype is actually overcompensated for by its involvement of the mitochondrial electron transport chain. The only extra chloroplastic ATP required in this PEP‐CK subtype arises from the regeneration of PEP by PPDK from malate decarboxylation‐derived pyruvate, which is 0.5 mol (if *n* = 3) or 0.572 mol (if *n* = 2.5) ATP per mole CO_2_ assimilated (Yin & Struik, 2018). Therefore, the proportion *x* of the chloroplastic ATP consumed by the C_4_ cycle is theoretically 0.15–0.17 in the PEP‐CK subtype, considerably lower than the 0.4 for the NADP‐ME and NAD‐ME subtypes (Table 1). Based on the NADPH and ATP budget, and assuming the same proportions of leakiness and photorespiration, Yin & Struik (2018) theoretically calculated that the $\Phi_{{\text{CO}}_{2}}$ of the PEP‐CK subtype is *c*. 0.080–0.081 mol mol^−1^ (Table 1), *c*. 26% higher than the $\Phi_{{\text{CO}}_{2}}$ of the NADP‐ME and NAD‐ME subtypes. Such an advantage, if engineered into C_4_ crops, would significantly increase canopy photosynthesis and, thus, crop yield. Indeed, the simulation of Yin & Struik (2017) showed that crop productivity can be increased almost linearly with decreasing ATP demands by the CCM.

However, literature‐reported values of $\Phi_{{\text{CO}}_{2}}$ measured under ambient conditions are not significantly higher in the PEP‐CK species than in C_4_ species of the other subtypes, especially not when compared with the NADP‐ME species (Ehleringer & Pearcy, 1983). The gaps between theoretically expected and experimentally measured $\Phi_{{\text{CO}}_{2}}$ in the PEP‐CK species have not been explained yet. Here, we first propose hypotheses that could explain the small advantage of the PEP‐CK subtype in $\Phi_{{\text{CO}}_{2}}$ over the other subtypes. On this basis, we describe a scheme for the energy budget in the PEP‐CK subtype and extend the model of Yin & Struik (2012) to calculate the $\Phi_{{\text{CO}}_{2}}$ of this PEP‐CK subtype. By exploring theoretical margins using this extended model, we propose a C_4_ ideotype and assess whether this would have advantages over the current crop C_4_ type (NADP‐ME) at the canopy level. With this approach, we hope to open a new horizon by redesigning strategies to introduce the C_4_‐ness into C_3_ crops, given that most attention so far has been paid to how to make C_4_ enzymes and structures function and little attention has been paid to the C_4_ energetics (Ermakova *et al*., 2020).

## Hypotheses

### Hypothesis 1: flux of amino groups between mesophyll and bundle‐sheath cells needs to be balanced

The classical PEP‐CK subtype (Hatch, 1987; Burnell & Hatch, 1988) was defined assuming that the ATP required by PEP‐CK could be exclusively produced by NADH oxidation in the respiratory chain of BS mitochondria associated with NAD‐ME‐dependent malate decarboxylation. If *n* = 3, only 25% of the CO_2_ from C_4_‐acid decarboxylation in BS cells is from the NAD‐ME activity; and if *n* = 2.5, the NAD‐ME‐associated decarboxylation will account for 28.6% of the CO_2_ generated. This definition of the PEP‐CK subtype did not consider the phosphate balance and the required amino (NH_2_)‐flux balance. Phosphate is generally believed to freely diffuse, and its availability is carefully regulated (Heldt *et al*., 1987). However, the NH_2_‐flux balance between M and BS cells is highly relevant for both NAD‐ME and PEP‐CK subtypes. In the NAD‐ME subtype, OAA reacts with glutamate (with NH_2_) to form aspartate, which is transported to BS mitochondria where it is deaminated to release OAA. The OAA is then converted to malate, and the malate decarboxylation product, pyruvate, is converted to alanine, which is shuttled to the M cytosol; thereby, the NH_2_ flux is balanced between M and BS cells (Fig. S1b). The direct OAA decarboxylation by PEP‐CK cannot achieve such a balance because its product, PEP, returns directly to the M cells (Fig. S1c). This inability to achieve the balance by direct OAA decarboxylation is one of the reasons why the ‘PEP‐CK‐only’ C_4_ type has hardly been identified in nature (Furbank, 2011). The parallel running of malate decarboxylation via NAD‐ME and direct OAA decarboxylation via PEP‐CK only alleviates the imbalance in the PEP‐CK subtype. The balance can be achieved only if the two decarboxylation pathways are equal in using the initial OAA (i.e. 50% each).

For such a scenario where NAD‐ME and PEP‐CK‐dependent decarboxylation each deliver 50% of CO_2_ to Rubisco, the required ATP per C_4_ cycle would be 1.5, consisting of 0.5 ATP for PEP‐CK activity in BS cells and 1.0 ATP for the regeneration of 0.5 mol PEP in M cells by PPDK. The oxidation of 0.5 NADH will provide 1.50 ATP (*n* = 3) or 1.25 ATP (*n* = 2.5), exactly or almost sufficient already to satisfy the required ATP for running the C_4_ cycle. However, the LET for supplying the extra 0.5 NADPH for this scenario also produces a certain amount of ATP, which can be calculated as $\left(2\times 0.5\right)\left(2+f_{Q}\right)/h$, where (2 + *f*_Q_)/*h* is ATP produced per LET (*f*_Q_, fraction of electrons at plastoquinone that follow the Q cycle; *h*, proton : ATP ratio) (Yin & Struik, 2012). Assuming the most likely values *f*_Q_ = 1 and *h* = 4 (Yin & Struik, 2012), the LET would produce 0.75 ATP, which is more than enough to fulfil the shortfall of 0.25 ATP if *n* = 2.5. The ‘50% each’ scenario was also proposed by Bräutigam *et al*. (2014) as the model for energy and NH_2_ balance of the PEP‐CK subtype. However, they overestimated the extra ATP requirement of this scenario because their analysis ignored that the NADH oxidation provides more ATP than required by the PEP‐CK activity and that the LET associated with 0.5 NADPH also produces ATP. Here, we indicate that, for this scenario, the CO_2_ assimilation of the PEP‐CK type would be NADPH limited (rather than ATP limited), and there is no need at all to engage the CET that is required for generating ATP in other C_4_ subtypes. The theoretical $\Phi_{{\text{CO}}_{2}}$ calculated using the approach of Yin & Struik (2018) for this type is 0.075 mol mol^−1^ (Table 1), which is somewhat lower than the theoretical $\Phi_{{\text{CO}}_{2}}$ (0.080–0.081 mol mol^−1^) for the classical PEP‐CK subtype but still higher than the measured $\Phi_{{\text{CO}}_{2}}$ of the PEP‐CK species.

### Hypothesis 2: PEP‐CK species represent various mixtures of the classically defined PEP‐CK component with NAD(P)‐ME and/or with less efficient PEP‐CK forms

Apparently, the aforementioned scenario of 50% each for two parallel decarboxylating pathways does not predict the measured $\Phi_{{\text{CO}}_{2}}$, nor can it generate the required balance in the ATP : NADPH ratio. We hypothesize that there must be a third, higher ATP‐requiring decarboxylating way (or even ways) involved in the PEP‐CK subtype.

First, there might be additional NAD‐ME or NADP‐ME‐based decarboxylation. Since there is hardly any NADP‐ME activity in PEP‐CK species (Hatch, 1987; Kanai & Edwards, 1999; Bräutigam, 2014; Koteyava *et al*., 2015), this third pathway likely involves NAD‐ME. The latter enzyme is already involved in the classical PEP‐CK subtype (associated with providing ATP for PEP‐CK activity); but we hypothesize that the additional NAD‐ME activity functions as it occurs in the classical NAD‐ME subtype, in which the NH_2_‐flux is balanced (see earlier). As such, on the one hand, NAD‐ME provides ATP for PEP‐CK activity and, on the other hand, functions independently with an increased ATP cost of CCM. However, the possibility that the third decarboxylation is NADP‐ME dependent or a mixed NAD‐ME and NADP‐ME type cannot be ruled out, as NADP‐ME has been found in some PEP‐CK species (Sonawane *et al*., 2018). Because the NADP‐ME itself does not involve any transamination, the NH_2_‐flux balance can also be guaranteed.

Second, the PEP produced by PEP‐CK in the BS cells is so far believed to move directly to the M cells (Fig. S1c). However, the fate of PEP is uncertain. The PEP can first be converted in BS cells to pyruvate, which moves via alanine aminotransferase to the M cells (Smith & Woolhouse, 1983), where pyruvate is converted to PEP by PPDK. The conversion of PEP to pyruvate in the BS cells could occur via pyruvate kinase, releasing one ATP per PEP converted (Schuler *et al*., 2016). Thus, the ATP balance for the C_4_ cycle is neutral in the BS cells as the one ATP required for PEP‐CK is equal to the one ATP released by pyruvate kinase. For this case, $\Phi_{{\text{CO}}_{2}}$ would be the same as for the first case where PEP‐CK is mixed with NAD‐ME or/and NADP‐ME.

Third, the conversion of PEP to pyruvate in BS cells could also occur via PEP phosphatase (Smith & Woolhouse, 1983). This enzyme releases orthophosphate, resulting in a loss of chemical energy to entropy (Bräutigam *et al*., 2018), in contrast to pyruvate kinase that conserves energy. Therefore, this case would predict an even lower $\Phi_{{\text{CO}}_{2}}$ in the PEP‐CK subtype than in the NADP‐ME and NAD‐ME subtypes, and works probably for the PEP‐CK species where $\Phi_{{\text{CO}}_{2}}$ is intrinsically low, or/and where only low activity of pyruvate kinase is detectable in BS cells (Kanai & Edwards, 1999).

The PEP in BS cells might also be converted via enolase and phosphoglyceromutase to 3‐phosphoglycerate (3‐PGA), which could move to the M cells and be converted back to PEP via the same enzymes (Huber & Edwards, 1975). This reaction may be engaged when the conversion of PEP to pyruvate cannot account for all of the carbon flux through the C_4_ cycle (Smith & Woolhouse, 1983). However, as this reaction does not involve any energy gain or loss, it would predict the same $\Phi_{{\text{CO}}_{2}}$ as the classical PEP‐CK type. Furthermore, the conversion to 3‐PGA cannot contribute to maintain the NH_2_‐flux balance. So, this mechanism will not be considered further. By contrast, the aforementioned second and third mechanisms involving pyruvate kinase and PEP phosphatase, respectively, not only can bring the theoretical $\Phi_{{\text{CO}}_{2}}$ of the PEP‐CK subtype equal to that of the ME subtypes but can also lower the BS : total ATP requirement ratio (compared with the previously discussed ‘pure PEP‐CK’ type) and maintain the NH_2_‐flux balance between M and BS cells. The high BS : total ATP requirement ratio (3 : 4, Yin & Struik, 2018) and the NH_2_‐flux imbalance (Furbank, 2011) were argued to explain why the ‘pure PEP‐CK’ type was hardly found in nature. Our hypothesis suggests that there are multiple scenarios for pure PEP‐CK and hints that a pure PEP‐CK type, if involving pyruvate kinase or PEP phosphatase, may actually exist.

## Model for the mixed PEP‐CK form

**Fig. 1:**
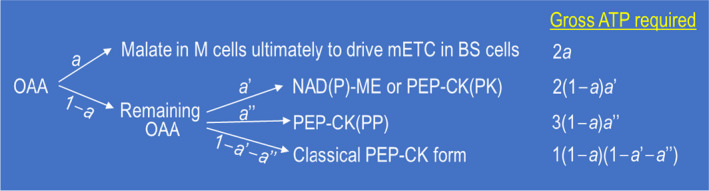
A scheme illustrating multiple decarboxylating pathways of C_4_photosynthesis. A fraction of OAA produced by phospho*enol*pyruvate (PEP) carboxylation in the mesophyll (M) cells, *a*, is reduced to malate, which will move to the bundle‐sheath (BS) mitochondria where malate is decarboxylated, generating NADH that will drive the mitochondrial electron transport chain (mETC) and produce ATP. The remaining fraction of OAA in the M cells, (1 − *a*), follows various possible pathways: a fraction of this part of OAA (*a*′) follows the NAD‐malic enzyme (ME) or NADP‐ME decarboxylating pathway or the PEP‐carboxykinase (CK) pathway where the PEP generated will be converted to pyruvate in the BS cells by pyruvate kinase (PK) – the pathway denoted as PEP‐CK(PK); another fraction (*a*″) follows the PEP‐CK pathway, where the PEP generated will be converted to pyruvate in the BS cells by PEP phosphatase (PP) – the pathway denoted as PEP‐CK(PP); and the remaining fraction, 1 − *a*′ − *a*″, follows the classical PEP‐CK pathway where the ATP required for PEP‐CK may come from the ATP generated from the aforementioned NADH‐associated mETC. As discussed in the main text, the NH_2_‐flux balance associated with the PEP‐CK type would require that *a* = (1 − *a*)(1 − *a*′ − *a*″). The gross ATP requirements for each decarboxylating form can be formulated according to these fractions, as indicated in the figure, where NAD‐ME, NADP‐ME, and PEP‐CK(PK) all require two ATP per decarboxylation (for PEP regeneration by pyruvate, phosphate dikinase), and therefore are lumped in the same category (but elsewhere in the text, and in Table 3 and Fig. 3, the fraction of this category for NADP‐ME is denoted as *b*). Summing individual gross ATP requirements up, deducting the ATP production by the NADH oxidation (which is *na*, where *n* is the number of ATP produced per NADH oxidation), would give the net ATP requirement for the whole C_4_cycle. Combining this with the NH_2_‐balance equation *a* = (1 − *a*)(1 − *a*′ − *a*″) and rearranging terms would give an equation describing net ATP requirement for the whole C_4_ cycle as 2 + *a*″ − (*n* + 1 +* a*″)*a* in this mixed type.

A scheme for quantitative proportions of possible decarboxylating pathways in the PEP‐CK subtype, which incorporates both Hypothesis 1 and Hypothesis 2, is provided in Fig. 1. This scheme both fulfils energetic demands and guarantees the NH_2_‐flux balance. The value of parameter *a* (the proportion of the initial OAA that is reduced to malate in M chloroplasts to ultimately drive mitochondrial electron transport in BS cells and produce ATP) is a crucial parameter for this mixed type. Its value has a strong impact on the cellular NADPH : ATP production ratio, and, thereby, on the required fraction for cyclic electron transport *f*_CET_. Both parameters, *a* and *f*_CET_, are hard to measure experimentally. We developed equations for calculating these two parameters using $\Phi_{{\text{CO}}_{2}}$ that can be easily measured. These equations and their derivations are given in Notes S1.

Note that photorespiration may be higher in species of the NAD‐ME or PEP‐CK subtypes than in the NADP‐ME subtype, because of larger amounts of photosystem II (PSII) in BS cells (thereby relatively more oxygen (O_2_) evolution as a result of LET), and because of decarboxylation taking place either in mitochondria or in cytosol (thereby, a higher chance for the released CO_2_ to escape from the refixation by Rubisco in BS chloroplasts, the organelle where decarboxylation occurs in the NADP‐ME subtype). Quantifying to what extent photorespiration is actually higher in the NAD‐ME, PEP‐CK, or mixed types relative to the exclusive NADP‐ME type is beyond the scope of this paper to quantify. To bypass this complication, we used our unpublished data from four PEP‐CK species on their $\Phi_{{\text{CO}}_{2}}$ measured using a gas mixture of 2% O_2_ and 1000 µmol mol^−1^ CO_2_; that is, under conditions in which photorespiration is conceivably negligible.

Values of $\Phi_{{\text{CO}}_{2}}$ (Table 2) were all lower than the theoretical value 0.080–0.081 mol mol^−1^ in Table 1 for the classically defined PEP‐CK subtype. But they were slightly higher than the values (0.060–0.067 mol mol^−1^) reported in the literature for this subtype (Ehleringer & Pearcy, 1983), suggesting that the gas mixture indeed prevented the occurrence of photorespiration. Since they are not lower than 0.064 mol mol^−1^, the theoretical $\Phi_{{\text{CO}}_{2}}$ for the ME subtypes, it is unlikely that PEP‐phosphatase‐based conversion of PEP into pyruvate in BS cells played a significant role, although we cannot rule it out. For simplicity, we set parameter *a*″ = 0 (see Fig. 1). Based on this value and other input parameter values that are considered conserved (Table S2), the calculated *a* depended slightly on the value of *n* and varied from 0 to 0.16, all lower than the minimum value for this parameter of 0.25 defining the classical PEP‐CK subtype. This means that, in order to meet the requirement of NH_2_‐flux balance, the fraction of OAA following the direct OAA decarboxylation by PEP‐CK was also from 0 to 0.16. The remaining dominant fraction (> 0.68) was to perform either NADP‐ME and NAD‐ME‐based decarboxylation, or the PEP‐CK decarboxylation involving pyruvate kinase. Though we were unable to quantitatively separate the three (but see later section), our calculations suggested that PEP‐CK species might have substantial NAD(P)‐ME activity. Bräutigam *et al*. (2014) did show that the NAD‐ME activity was 35% higher than the PEP‐CK activity in the PEP‐CK species *Panicum maximum*. However, the current literature data on the activities of the three decarboxylating enzymes in the PEP‐CK species (e.g. Hatch, 1987; Kanai & Edwards, 1999; Koteyava *et al*., 2015; Bräutigam *et al*., 2018; Sonawane *et al*., 2018) suggest that generally PEP‐CK is still dominant over the other two decarboxylating enzymes. So, most likely, the PEP‐CK decarboxylation involving pyruvate kinase accounts for the third source of CO_2_ delivered to Rubisco.

**Table 2:** Values of parameters *a* and *f*_CET_, calculated from unpublished estimates of $\Phi_{{\text{CO}}_{2}}$ (SEs of estimates in parentheses) from measurements for four phospho*enol*pyruvate carboxykinase species.

Species	$\Phi_{{\text{CO}}_{2}\text{(NPR)}}$ (mol mol^−1^)	*n* = 3		*n* = 2.5	
		*a*	*f*_CET_	*a*	*f*_CET_
*Chloris gayana*	0.068 (0.003)	0.01	0.509	0.01	0.508
*Melinis minutiflora*	0.065 (0.004)	0.00	0.517	0.00	0.517
*Panicum maximum*	0.072 (0.003)	0.13	0.386	0.16	0.373
*Spartina gracilis*	0.069 (0.004)	0.05	0.479	0.05	0.475

The calculation assumed that *ϕ* (leakiness) = 0.16; *n* = ATP : NADP ratio.

*a*, proportion of the initial oxaloacetate for converting to malate in mesophyll cells that ultimately leads to mitochondrial electron transport in bundle‐sheath cells to produce ATP; *f*_CET_, fraction for cyclic electron transport.

Measurements for estimating $\Phi_{{\text{CO}}_{2}\text{(NPR)}}$ were conducted on leaves of four replicated plants for each species, using a gas mixture of 2% oxygen combined with 1000 µmol mol^−1^ CO_2_, with which a nearly nonphotorespiratory (NPR) condition is conceivably achieved.

## Exploring theoretical margins to design an ideotype of C_4_ photosynthesis

**Fig. 2:**
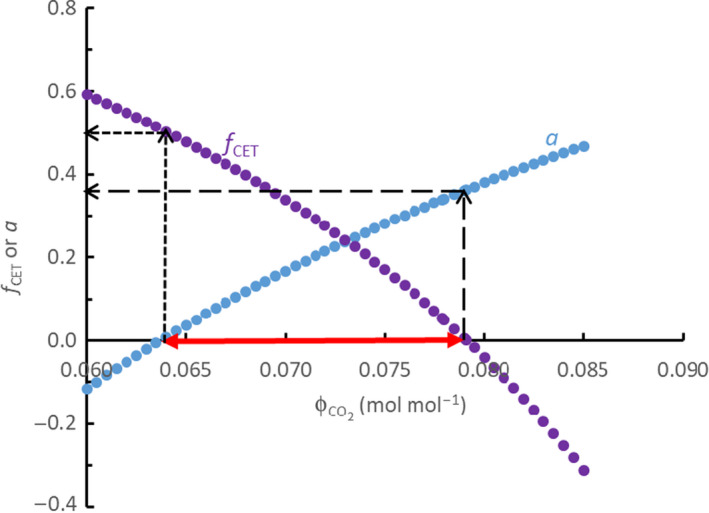
Exploring theoretical margins of the proposed mixed phospho*enol* pyruvate carboxykinase type to identify the optimum combination of parameters *a* (proportion of OAA reduced to malate in mesophyll cells for malate decarboxylation via NAD‐malic enzyme (ME) to drive mitochondrial electron transport in bundle‐sheath cells) and *f*_CET_ (fraction for cyclic electron transport) that maximizes $\Phi_{{\text{CO}}_{2}}$within the physiologically relevant range of *a* and *f*_CET_ indicated by the thick red arrow; that is, the range when both are ≥ 0. The maximum $\Phi_{{\text{CO}}_{2}}$is achieved when *f*_CET_ = 0, and its equivalent value of parameter *a* in this illustration (where *n*, the ATP : NADH ratio, is set to 3.0) is *c*. 0.363, indicated by long‐dashed black arrows. When *a* = 0, *f*_CET_ ≈ 0.5 and $\Phi_{{\text{CO}}_{2}}\approx \;0.064$ mol mol^−1^, as indicated by short‐dashed black arrows; this case corresponds to the classical NADP‐ME subtype or NAD‐ME subtype.

The model described in Notes S1 for calculating parameters *a* and *f*_CET_ can be used to explore theoretical margins for the optimum combination of *a* and *f*_CET_ that gives the maximum $\Phi_{{\text{CO}}_{2}}$ within the physiologically relevant range of *a* and *f*_CET_. This is illustrated in Fig. 2, where the physiologically relevant range is marked by the red arrow. The lowest $\Phi_{{\text{CO}}_{2}}$ of this range is defined by *a* = 0, which means that the mixed type would become the classically defined NADP‐ME or NAD‐ME subtype. The maximum $\Phi_{{\text{CO}}_{2}}$ of this range is defined by *f*_CET_ = 0, which gives the most efficient mixed type having the maximum $\Phi_{{\text{CO}}_{2}}$, and the equivalent value of parameter *a* ≈ 0.36 if *n* = 3 (Fig. 2) or *a* ≈ 0.40 if *n* = 2.5. Beyond this point, *f*_CET_ would become negative. The NH_2_‐flux balanced PEP‐CK type (where *a* = 0.5) discussed earlier actually goes beyond this threshold that would need a negative *f*_CET_ ≈ −0.44 (if *n* = 3) or *f*_CET_ ≈ −0.26 (if *n* = 2.5). The logic for negative *f*_CET_ is that, with parameter *a* becoming higher, NADPH in the M cell is overused for reducing OAA to malate such that NADH oxidation and LET are both high, overproducing ATP that would need a negative *f*_CET_ to balance the NADPH : ATP ratio. As a negative *f*_CET_ is obviously physiologically impossible, the NH_2_‐flux balanced PEP‐CK type (where *a* = 0.5) discussed earlier should not exist in nature.

The maximum $\Phi_{{\text{CO}}_{2}}$ at *f*_CET_ = 0 was 0.079 mol mol^−1^ (if *n* = 3) or 0.078 mol mol^−1^ (*n* = 2.5), only slightly lower than $\Phi_{{\text{CO}}_{2}}$ of 0.080–0.081 mol mol^−1^, the value calculated for the classically defined PEP‐CK subtype (Table 1). However, as discussed earlier, the classically defined PEP‐CK subtype does not meet the requirement for a balance of the NH_2_‐flux between M and BS cells. As a result, unlike the classically defined NADP‐ME or NAD‐ME subtypes (which are special cases when *a* = 0 in Fig. 2), the classically defined PEP‐CK subtype cannot be a special case along the curves shown in Fig. 2. As the type with *f*_CET_ = 0 (Fig. 2) yields the highest $\Phi_{{\text{CO}}_{2}}$, we shall call it the C_4_ ideotype. This ideotype, potentially as a mixture of three C_4_ subtypes, guarantees a balanced NH_2_‐flux and a balanced NADPH : ATP ratio as required by C_3_ and C_4_ cycles.

## Can energy supply satisfy cell‐type‐specific energy demand in the designed ideotype?

It is necessary to examine if NADPH and ATP requirements can be met within M and BS cell types. To that end, we extended the cell‐type‐specific models of Yin & Struik (2018) for light absorption, energy production, and requirement for the mixed C_4_ type as defined in Fig. 1 (Notes S2). We run this extended NADPH and ATP production model for the C_4_ ideotype with *a* = 0.36 (if *n* = 3) or *a* = 0.40 (if *n* = 2.5) and *a*″ = 0, using four sets of the three input parameters: leaf Chl content [CHL], fraction of [CHL] in BS cells, and fraction of photosystem I (PSI) in BS cells. These four sets of inputs were taken from measurements of Ghannoum *et al*. (2005) for two NADP‐ME (sets I and II) and two NAD‐ME (sets III and IV) species. The model can identify the physiologically relevant fraction of PSII in BS cells (Yin & Struik, 2018). Other output parameters relevant for our analysis here are the fraction of ATP production in BS cells and the fraction of NADPH production in BS cells. None of these three output parameters depended on the value of *n*, so they are given in a single value for each input set (Table 3). The model identified that the physiologically relevant fraction of PSII in BS cells had a single value and was equal to the input fraction of PSI in BS cells. This is because the proposed ideotype requires no CET; so, relative amounts of PSII in either cell type should agree with the relative amounts of PSI to perform LET only. For the same reason, the predicted fraction of ATP production in BS cells equalled the predicted fraction of NADPH production in BS cells (Table 3), as LET produces a fixed ATP : NADPH ratio.

**Table 3:** Modelled output parameter values of the proposed C_4_ ideotype assuming the ATP : NADH ratio *n* = 3 (before ‘/’ sign) or = 2.5 (after ‘/’ sign), using four sets (I–IV) of three input‐parameter values.

Parameter		I	II	III	IV
Input	[CHL] (µmol m^−2^)	579	464	424	425
	Fraction of [CHL] in BS	0.33	0.38	0.60	0.59
	Fraction of PSI in BS	0.37	0.39	0.24	0.46
Output	Fraction of PSII in BS (*α*)	0.37	0.39	0.24	0.46
	Fraction of ATP produced in BS^a^	0.29	0.32	0.48	0.47
	Fraction of NADPH produced in BS^a^	0.29	0.32	0.48	0.47
	*γ*_atp_ ^b^	0.35/0.29	0.42/0.36	0.73/0.67	0.71/0.65
	*b* ^c^	−0.04/−0.65	0.13/−0.43	0.79/0.48	0.75/0.44
	NADP‐ME% in total decarboxylation^d^	−1.0/−13.2	3.4/−8.7	21.5/9.7	20.6/8.8
	*γ*_nadph_ (if *b* = 0)^e^	0.35/0.36	0.40/0.41	0.61/0.61	0.60/0.60

^a^ Cell‐type‐specific production of ATP and NADPH quantified as the bundle‐sheath (BS) : total ratio.

^b^ *γ*_atp_, fraction of 3‐phosphoglycerate (3‐PGA) reduction in BS cells inferred from the balance that the BS : total ratio in ATP production is equal to the BS : total ratio in ATP demand.

^c^ *b*, the fraction of the second decarboxylation category (see Fig. 1) that is via NADP‐malic enzyme (ME), inferred from assuming that *γ*_nadph_ equals *γ*_atp_.

^d^ Percentage of the NADP‐ME pathway accounting for the total decarboxylation, estimated as [1 − 2*a* − (1 − *a*)*a*″]*b* × 100 (see Supporting Information Notes S2), where *a* = 0.36 (if *n* = 3) or 0.40 (if *n* = 2.5), and *a*″ = 0 (see the text).

^e^ *γ*_nadph_, fraction of 3‐PGA reduction in BS cells inferred from the balance that the BS : total ratio in NADPH production is equal to the BS : total ratio in NADPH demand.

We then analysed whether ATP and NADPH supply satisfies cell‐type‐specific energy requirement in the C_4_ ideotype, following the same method as used by Yin & Struik (2018); that is, by matching the fraction of ATP (NADPH) supply in BS cells with the fraction of ATP (NADPH) demand in BS cells. The formulae for the cell‐type‐specific demands are described in Table S3 (where *a*″ should be set to zero for the ideotype). This matching gives rise to what fraction of the 3‐PGA reduction in BS cells would be needed. Table S3 shows that the cell‐type‐specific ATP demand does not depend on parameter *b* (fraction of the second category of decarboxylation in Fig. 1 that is via NADP‐ME) whereas the NADPH demand does. So, we first calculated the required fraction of the 3‐PGA reduction in BS cells to have the ATP supply and demand in balance, indicated by the variable *γ*_atp_. It varied from 0.35 to 0.73 when *n* = 3 or from 0.29 to 0.67 when *n* = 2.5 (Table 3). The higher *γ*_atp_ when *n* is higher was because higher ATP supply from NADH oxidation in BS mitochondria can support a higher fraction of 3‐PGA reduction in BS chloroplasts; and the transfer of ATP can be realized given available intracellular ATP shuttles (Shameer *et al*., 2019). The significantly higher fraction of 3‐PGA reduction in BS cells in sets III and IV than in sets I and II was due to higher fractions of [CHL] in BS cells in sets III and IV, which enabled the capture of relatively more light energy and therefore to produce relatively more ATP in BS cells (Table 3).

Assuming that *γ*_nadph_ (fraction of the 3‐PGA reduction in BS cells to have the NADPH supply and demand in balance) equalled *γ*_atp_, we were able to calculate *b*, which varied from −0.04 to 0.79 when *n* = 3 or from −0.65 to 0.48 when *n* = 2.5, and the resulting percentage of the NADP‐ME pathway accounting for the total decarboxylation was also calculated (Table 3). The higher *b* when *n* = 3 was because this *n* resulted in higher *γ*_atp_, which, when setting to be *γ*_nadph_, would result in higher requirements for shuttling malate from M to BS cells by the NADP‐ME‐dependent pathway. Negative values of *b* and NADP‐ME%, as occurred for sets I and II (Table 3), are physiologically impossible; their occurrence merely suggests that the input parameter value for the fraction of [CHL] in BS cells in set I and probably also in set II was not sufficiently high to realize the proposed C_4_ ideotype. Assuming *b* = 0, we recalculated *γ*_nadph_. Compared with *γ*_atp_, the *γ*_nadph_ obtained was higher in cases where *b* was earlier found to be less than zero, but lower in cases where *b* was greater than zero (Table 3). 3‐PGA as the product of RuBP carboxylation is produced in BS cells. Its reduction involves an intercellular shuttle (Hatch, 1987), during which 3‐PGA is first transformed to 1,3‐bisphosphoglycerate by phosphoglycerate kinase (the step that requires ATP) and then reduced to triose phosphate by glyceraldehyde 3‐phosphate dehydrogenase (the step that requires NADPH). Higher *γ*_atp_ than *γ*_nadph_, as calculated for sets III and IV, indicates the possibility that, relative to the second step, the first step occurs more in BS than in M cells. However, it is impossible to reconcile with a higher *γ*_nadph_ than *γ*_atp_, as calculated mostly for sets I and II, again suggesting that the proposed C_4_ ideotype cannot be achieved with a low [CHL] fraction in BS cells.

Our model analysis herein suggests that cellular energy production can satisfy cell‐type‐specific ATP and NADPH requirement in the C_4_ ideotype, provided that the fraction [CHL] in BS cells is greater than *c*. 0.4 and the proportion of 3‐PGA reduction in BS cells and, optionally, the proportion of NADP‐ME operation can adjust accordingly. The latter suggests an approach to estimate a maximum NADP‐ME% in total decarboxylation (Table 3).

## Advantages of the C_4_ ideotype over the NADP‐ME type at the canopy level of a crop

Finally, we evaluated whether the proposed C_4_ ideotype can indeed have advantages over the current crop C_4_ type (NADP‐ME subtype) in a canopy and crop setting. To evaluate that, we run the crop model Gecros (Yin & Struik, 2017), for which we revised its C_4_ submodel based on the algorithms developed here for the proposed C_4_ ideotype (see Notes S3). We set the fraction of PSII in BS cells *α* = 0.10 for the crop C_4_ type as observed in maize and *α* = 0.40 for the C_4_ ideotype, the general value for the PEP‐CK subtype (von Caemmerer & Furbank, 1999). We used the 31 yr (1980–2010) weather data of the International Rice Research Institute (in the Philippines), with Gecros input crop parameters as described by Yin & Struik (2017).

The simulated 31 yr average advantage of the crop type C_4_ (NADP‐ME) over the C_3_ rice in radiation use efficiency was 36.3%, and the advantage of the proposed C_4_ ideotype was *c*. 50% when *n* = 3 and *c*. 48% when *n* = 2.5 (Table 4). Simulated advantages in total biomass all became somewhat higher, because an increased leaf area index was simulated as a consequence of the increased photosynthesis or radiation use efficiency, resulting in somewhat higher light interception during growing seasons. The advantage of the crop C_4_ type was 42%, whereas that of the proposed C_4_ ideotype was always >50% (Table 4). The required increase of *α* in the ideotype relative to the NADP‐ME subtype is expected to increase photorespiration, but the impact of varying *α* on simulated advantages was small (results not shown). That was probably because, in the simulation, we assumed that the BS conductance (that determines the CO_2_ leakage) in the proposed C_4_ ideotype was as small as that found for the NADP‐ME species, where decarboxylation occurs predominantly in chloroplasts. Whether or not this conductance becomes higher in the NAD‐ME (decarboxylation in mitochondria) subtype and the PEP‐CK subtype (decarboxylation in cytosol and mitochondria) is an important subject to investigate, although PEP‐CK has been expressed to localize in chloroplasts of transgenic rice plants (Suzuki *et al*., 2000).

**Table 4:** Simulated advantages (%) of the current crop C_4_ type (the NADP‐malic enzyme (ME) subtype) and the proposed C_4_ ideotype over the C_3_ rice in radiation use efficiency and total biomass production.

		Radiation use efficiency	Total biomass
C_3_ rice		2.95 g DM MJ^−1^ PAR	22.6 t DM ha^−1^
C_4_	NADP‐ME C_4_ type (*α* = 0.1)	36.3%	42.0%
	C_4_ ideotype (*α* = 0.4)	49.7%/47.5%	55.2%/52.7%

PAR, photosynthetically active radiation.

Radiation use efficiency is the ratio of total biomass to season‐long accumulated PAR interception.

*α*, fraction of photosystem II in bundle‐sheath cells.

For the C_4_ ideotype, the percentages before and after the ‘/’ sign refer to the simulated advantages using *n* = 3 or *n* = 2.5, respectively (*n*, the number of ATP formed per NADH oxidation; see the text).

## Concluding remarks

C_4_ crops of agricultural importance all belong to the NADP‐ME subtype. Whether there is any causality behind this or whether it is just the coincidence of domestication is an interesting question to investigate. However, operating the CCM cycle in either NADP‐ME or NAD‐ME subtypes is energetically expensive, accounting for at least 40% of total ATP cost for CO_2_ assimilation. Yin & Struik (2017) showed that the crop C_4_ type, if being introduced into C_3_ crops, would not be able to increase yield by 50%, the initially expected yield advantage of C_4_ rice (Mitchell & Sheehy, 2006). We therefore proposed a C_4_ ideotype by exploring theoretically lower energy requirements in the nondomesticated PEP‐CK subtype.

**Fig. 3:**
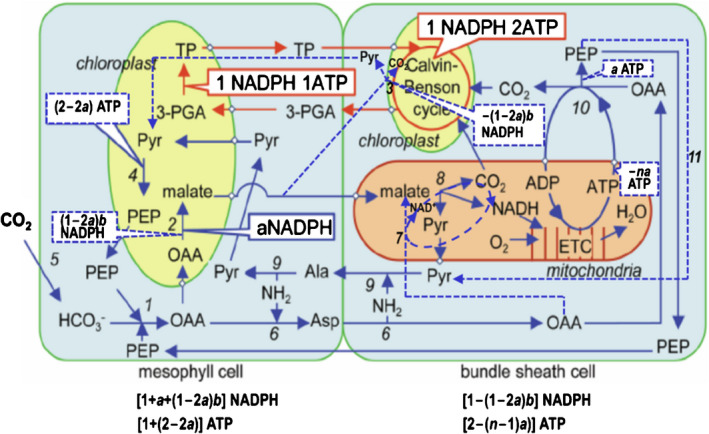
The proposed mixed type of C_4_ photosynthesis, and its NADPH and ATP requirements per CO_2_ assimilation in mesophyll (M) and bundle‐sheath (BS) cells. The red arrows show the Calvin cycle and its cell‐type‐specific NADPH and ATP requirements; the blue arrows show the C_4_ cycle, where the full arrows show the cycle in the classically defined phospho*enol* pyruvate (PEP)‐carboxykinase (CK) subtype (see Supporting Information Fig. S1c; reprinted by permission from Springer Nature, Ishikawa *et al*., 2016) and the dashed ones show the new components added to form the mixed type. For illustrative simplicity, it is assumed that there is no leakiness, that the 3‐phosphoglycerate (3‐PGA) reduction of the Calvin cycle occurs in 50% in each of the two cell types, and that the PEP‐CK pathway where the PEP generated is converted to pyruvate in the BS cells possibly by PEP phosphatase (PP) – the pathway denoted as PEP‐CK(PP) in Fig. 1 – does not occur (for detailed algorithms for energy requirement without these assumptions, see Table S3). The coefficient *a* refers to the fraction of OAA produced by PEP carboxylation in the M cells that is reduced to malate moving to the BS mitochondria where malate decarboxylation generates NADH that will drive mitochondrial electron transport to produce ATP. This produces *na* ATP (where *n* is the ATP : NADH ratio). The coefficient *b* is the fraction of the second decarboxylation category defined in Fig. 1that belongs to the NADP‐malic enzyme (ME) type. If *a* = 0 and *b* = 1, then the mixed type becomes the NADP‐ME subtype (see Fig. S1a); if *a* = 0 and *b* = 0, then the mixed type becomes the NAD‐ME subtype (see Fig. S1b). Another special case if *a* = 0 and *b* = 0 is a hypothetical ‘pure’ PEP‐CK type, where the PEP generated will be converted to pyruvate in the BS cells by pyruvate kinase – the pathway denoted as PEP‐CK(PK) in Fig. 1. Numbers represent enzymes involved in the C_4_ cycle: 1, PEP carboxylase (PEPc); 2, NADP‐malate dehydrogenase (NADP‐MDH); 3, NADP‐ME; 4, pyruvate, phosphate dikinase (PPDK); 5, carbonic anhydrase (CA); 6, aspartate aminotransferase (AspAT); 7, NAD‐malate dehydrogenase (NAD‐MDH); 8, NAD‐ME; 9, alanine aminotransferase (AlaAT); 10, PEP‐CK; 11, pyruvate kinase (PK). Ala, alanine; Asp, aspartate; ETC, electron transport chain; Pyr, pyruvate; TP, triose phosphate.

The ideotype is the special form of the general mixed NAD(P)‐ME and PEP‐CK (Fig. 1) that results in the maximum $\Phi_{{\text{CO}}_{2}}$ (Fig. 2). The general mixed type considers the balance of the NH_2_ groups between M and BS cells and the balance of NADPH and ATP as the metabolically required ratio. The classically defined PEP‐CK subtype is not a special form of our general type, because it does not satisfy the first balance; neither is the NH_2_‐flux balanced type discussed earlier, as it does not satisfy the second balance. However, the other two classical subtypes are special cases of the general type. As species traditionally classified as NADP‐ME or NAD‐ME types often have some PEP‐CK (Furbank, 2011), which can even be induced to increase by environmental changes (e.g. Sales *et al*., 2018), our general type may be considered as a unified scheme of C_4_ subtypes (Fig. 3).

The proposed C_4_ ideotype does not need CET under low irradiances; so, probably few modifications of electron transport machinery are required because C_3_ photosynthesis involves little CET. Furthermore, the PEP‐CK is present in C_3_ plants although it is responsible for nonphotosynthetic functions (Leegood & Walker, 2003), and genes encoding the evolution of C_4_ PEP‐CK have been reported (Christin *et al*., 2009). However, the required engagement of multiple biochemical pathways (Fig. 3) might pose a challenge to engineering. Moreover, the required proportion of [CHL] in BS cells needs to be higher than that found in current C_4_ crop species, and the vein spacing in C_3_ leaves may not allow the housing of sufficient chloroplasts in BS cells (Karki *et al*., 2013; Ermakova *et al*., 2020). Wang *et al*. (2017) reported that transgenic rice lines expressing *Golden‐like* transcription factor genes from maize displayed increased numbers and volumes of differentiated chloroplasts, and accumulated more [CHL], in BS and mestome sheath cells of the leaf. As long as the required [CHL] in BS cells can be reached, there are a range of options to achieve cellular energy balance of both ATP and NADPH; for example, via adjusted proportion of 3‐PGA reduction in two cell types and adjusted engagement of the NADP‐ME pathway (Table 3).

The required value of parameter *a* (proportion of OAA reduced to malate in M cells for malate decarboxylation via NAD‐ME to drive mitochondrial electron transport) for the C_4_ ideotype was *c*. 0.36 or 0.40. This is higher than the 0.25 or 0.286 required for the classically defined PEP‐CK subtype (Hatch, 1987; Kanai & Edwards, 1999; Ishikawa *et al*., 2016). As stated, the classical PEP‐CK type does not meet the NH_2_ balance and, thus, should not exist in nature. We do not know yet if there are C_4_ species in nature that are physiologically the same as, or close to, the defined C_4_ ideotype. Carnal *et al*. (1993) reported that maximum rates of malate decarboxylation via NAD‐ME substantially exceeded the minimum rates necessary for providing ATP for cytosolic OAA decarboxylation by PEP‐CK. This suggests that PEP‐CK species having high proportions (close to 0.36 or 0.40) of OAA reduced to malate in M cells for malate decarboxylation via NAD‐ME may actually exist, although they may not necessarily have a high $\Phi_{{\text{CO}}_{2}}$. Reasons for the low $\Phi_{{\text{CO}}_{2}}$ in PEP‐CK species need to be examined in conjunction with our aforementioned hypotheses.

Photosynthetic $\Phi_{{\text{CO}}_{2}}$ is known to be recalcitrant to genetic improvement in both C_3_ and C_4_ types. However, canopy photosynthesis after canopy closure generally depends more on $\Phi_{{\text{CO}}_{2}}$ than on *A*_max_ (Day & Chalabi, 1988; Gu *et al*., 2014), especially given recent molecular evidence that electron transport is a major limitation to photosynthesis in C_4_ plants (Ermakova *et al*., 2019). Our viewpoints provide a (theoretical) case where $\Phi_{{\text{CO}}_{2}}$ could be improved, thereby potentially paving a new way of redesigning and supercharging photosynthesis. If this is proven either from exploiting natural variations or by a synthetic biology approach, it would provide a new C_4_ template not only for being introduced into C_3_ crops but also for improving current C_4_ crops.

## Author contributions

XY conceived the viewpoints and designed the modelling. XY wrote the draft and finalized it with significant input from PCS.

## Supporting information


**Fig. S1** The three classically‐defined C_4_ subtypes classified according to decarboxylation enzymes.
**Notes S1** Deriving the equation for quantum yield for CO_2_‐assimilation (Φ_CO2_), and equations for calculating parameters a and f_CET_ from the measured Φ_CO2_.
**Notes S2** Extending the model of Yin & Struik (2018) to accommodate the mixed type.
**Notes S3** Extending the C_4_ submodel in crop model GECROS to accommodate the C_4_ ideotype.
**Table S1** Definitions and units of model symbols.
**Table S2** Indicative values of model input parameters used in the analysis.
**Table S3** Formulae for calculating cell‐type‐specific NADPH and ATP demands per CO_2_ assimilation in the mixed type.
Please note: Wiley Blackwell are not responsible for the content or functionality of any Supporting Information supplied by the authors. Any queries (other than missing material) should be directed to the *New Phytologist* Central Office.
