# Supplementary material for: Exploiting differences in the energy budget among C4 subtypes to improve crop productivity
Source: New Phytol. 2020 Nov 20;229(5):2400–9. doi: 10.1111/nph.17011 (PMC7894359; doi:10.1111/nph.17011)
Supplement: Supplementary file 1 — Fig. S1 The three classically‐defined C4 subtypes classified according to decarboxylation enzymes. Notes S1 Deriving the equation for quantum yield for CO2‐assimilation (ΦCO2), and equations for calculating parameters a and fCET from the measured ΦCO2. Notes S2 Extending the model of Yin & Struik (2018) to accommodate the mixed type. Notes S3 Extending the C4 submodel in crop model GECROS to accommodate the C4 ideotype. Table S1 Definitions and units of model symbols. Table S2 Indicative values of model input parameters used in the analysis. Table S3 Formulae for calculating cell‐type‐specific NADPH and ATP demands per CO2 assimilation in the mixed type. Please note: Wiley Blackwell are not responsible for the content or functionality of any Supporting Information supplied by the authors. Any queries (other than missing material) should be directed to the New Phytologist Central Office. [file NPH-229-2400-s001.pdf]

New Phytologist

New Phytologist Supporting Information

Article title: Exploiting differences in the energy budget among C_4 subtypes to improve crop productivity
Authors: Xinyou Yin & Paul C. Struik
Article acceptance date: 11 October 2020

The following Supporting Information is available for this article:

Fig. S1 The three classically-defined C_4 subtypes classified according to decarboxylation enzymes

Notes S1 Deriving the equation for quantum yield for CO_2-assimilation (Φ_{CO2}), and equations for calculating parameters *a* and f_{CET} from the measured Φ_{CO2}

Notes S2 Extending the model of Yin & Struik (2018) to accommodate the proposed mixed type

Notes S3 Extending the C_4 submodel in crop model GECROS to accommodate the C_4 ideotype

Table S1 Definitions and units of model symbols

Table S2 Indicative values of model input parameters used in the analysis

Table S3 Formulae for calculating cell-type-specific NADPH and ATP demands per CO_2 assimilation in the mixed PEP-CK type proposed in Fig. 1

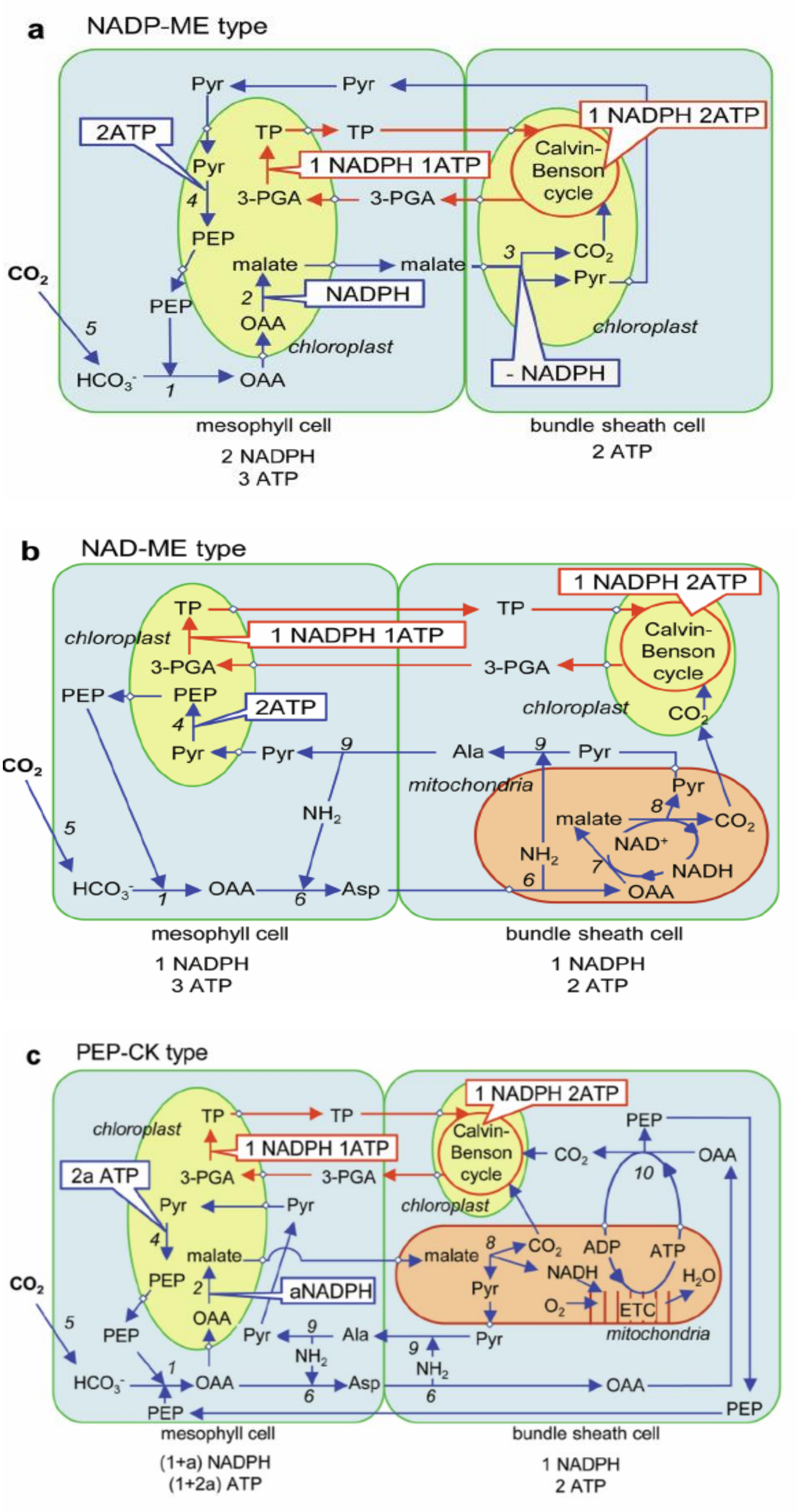

Figure S1 The three classically-defined C_4 subtypes classified according to decarboxylation enzymes (a, NADP-ME; b, NAD-ME; and c, PEP-CK), and their minimum cell-type specific energy requirements assuming (i) no leakiness, (ii) 50% of the 3-PGA reduction takes place in M and BS cells each, and (iii) no photorespiration and alternative electron and ATP sinks (Reprinted by permission from Springer Nature, Ishikawa et al. 2016). Abbreviations and numbers are as given in Fig. 3.

Notes S1 Deriving the equation for quantum yield for CO_2-assimilation (Φ_{CO2}), and equations for calculating parameters a and f_{CET} from the measured Φ_{CO2}

The model described below for the general mixed type as described in Fig. 1 is based on the model of Yin & Struik (2012) for electron-transported limited C_4 photosynthesis in the NADP-ME and NAD-ME subtypes.

For any type of (C_3 or C_4) photosynthesis when involving two types of electron transport: linear and cyclic electron transport (LET and CET, respectively), the factor for excitation partitioning to PSII (ρ_2; see Table S1 for symbol definitions) can be expressed as (Yin & Struik 2012, their eqn A5):

$$\rho_2 = \frac{1-f_{\mathrm{CET}}}{1-f_{\mathrm{CET}}+r_{2/1}} \quad (1.1)$$

where f_{CET} is the fraction of total PSI electrons that follow CET, and $r_{2/1}$ is the PSII : PSI electron transport efficiency ratio.

Quantum yield of CO_2 assimilation in terms of NADPH

Total NADPH production rate (J_{nadph}) is

$$J_{\mathrm{nadph}} = 0.5\rho_2 I_{\mathrm{abs}}\Phi_2 \quad (1.2)$$

where I_{abs} is absorbed irradiance, $\rho_2 I_{abs}\Phi_2$ as a whole is the rate of LET, and 0.5 stands for mol NADPH produced per LET. NADPH demand per mol CO_2 assimilation, d_{nadph}, is

$$d_{\mathrm{nadph}} = 2 + 2v_{\mathrm{o/c}} + 5v_{\mathrm{n/c}} + a(1+\phi) \quad (1.3)$$

where $v_{o/c}$ is the RuBP oxygenation : carboxylation ratio, $v_{n/c}$ is the nitrogen assimilation : RuBP carboxylation ratio, ϕ is leakiness, and a is the term specific for any type involving PEP-CK as defined in Fig. 1 (i.e. the fraction of OAA that is reduced to malate using the NADPH of M cells for shuttling to BS cells to drive mitochondrial electron transport). Eqn (1.3) assumes that the process (other than RuBP carboxylation and oxygenation) that uses electrons of LET is predominantly nitrate reduction, requiring 10 mol electrons (equivalent to 5 mol NADPH) per nitrate reduction (Noctor & Foyer 1998). Note that electron requirement for nitrate reduction was lumped to the pseudocyclic electron fraction (f_{pseudo}) in the model of Yin & Struik (2012).

Combining eqns (1.1-1.3) and considering photorespiratory CO_2 release give the equation for gross CO_2 assimilation rate in terms of NADPH ($A_{g,nadph}$)

$$A_{\mathrm{g,nadph}} = \left(1-0.5v_{\mathrm{o/c}}\right)\frac{J_{\mathrm{nadph}}}{d_{\mathrm{nadph}}} = \frac{(1-0.5v_{\mathrm{o/c}})\frac{1-f_{\mathrm{CET}}}{1-f_{\mathrm{CET}}+r_{2/1}}\Phi_2 I_{\mathrm{abs}}}{4+4v_{\mathrm{o/c}}+10v_{\mathrm{n/c}}+2a(1+\phi)} \quad (1.4)$$

Equation (1.4) assumes 0.5 mol CO_2 is produced per oxygenation (Farquhar et al. 1980). Quantum yield of CO_2 assimilation in terms of NADPH ($\Phi_{CO2,nadph}$) would be

$$\Phi_{\mathrm{CO2,nadph}} = \frac{A_{\mathrm{g,nadph}}}{I_{\mathrm{abs}}} = \frac{(1-0.5v_{\mathrm{o/c}})(1-f_{\mathrm{CET}})\Phi_2}{(1-f_{\mathrm{CET}}+r_{2/1})[4+4v_{\mathrm{o/c}}+10v_{\mathrm{n/c}}+2a(1+\phi)]} \quad (1.5)$$

Quantum yield of CO_2 assimilation in terms of ATP

Similar logic can be used to define equation ATP-dependent quantum yield of CO_2 assimilation but this has more uncertain parameters. Total ATP production rate (J_{atp}) is

$$J_{\mathrm{atp}} = z(\rho_2 I_{\mathrm{abs}})\Phi_2 = \frac{2+f_{\mathrm{Q}}-f_{\mathrm{CET}}(1-2f_{\mathrm{NDH}})}{h(1-f_{\mathrm{CET}})}(\rho_2 I_{\mathrm{abs}})\Phi_2 \quad (1.6)$$

where *z* is the factor of ATP production per LET when CET runs simultaneously, in which f_Q is the fraction of electrons at plastoquinone that follow the Q-cycle (= 1 for C_4 photosynthesis, Furbank et al. 1990), *h* is protons required per ATP synthesis (either 4 or 14/3), and f_{NDH} is the fraction of CET that follows the NAD(P)H dehydrogenase (NDH)-dependent pathway. Uncertainties exist with regard to whether f_{NDH} should be included in the model, but here we include f_{NDH} so that our model covers all scenarios (whereby the *z* factor was derived following the same procedure as described by Yin et al. 2004). If $h = 4$ (as identified by thermodynamic experimental calculations, Steigmiller et al. 2008; Petersen et al. 2012), then the NDH-dependent pathway is not needed (i.e. $f_{NDH} = 0$; then the expression for *z* in eqn (1.6) becomes eqn (1) of Yin & Struik 2012). However, if $h = 4.67$ (14/3; as identified by structural data for the c14 rotor ring of the proton translocating chloroplast ATP synthase (Seelert et al. 2000), then f_{NDH} would be needed.

The ATP demand per mol CO_2 assimilation, d_{atp}, is

$$d_{\mathrm{atp}} = 3 + 3.5v_{\mathrm{o/c}} + 1v_{\mathrm{n/c}} + c_{\mathrm{starch}}(1-0.5v_{\mathrm{o/c}} - v_{\mathrm{r/c}}) + [2 + a'' - (n + 1 + a'')a](1+\phi) \quad (1.7)$$

where c_{starch} is ATP cost per carbon in starch synthesis (=0.167; Noctor & Foyer 1998), $v_{r/c}$ is day respiration to RuBP carboxylation ratio, and $[2+a''-(n+1+a'')a]$ is the net chloroplast ATP requirement to operate the C_4 cycle for the mixed type (see Fig. 1). Eqn (1.7) assumes that 1 mol ATP per nitrate reduction (Noctor & Foyer 1998) comes from chloroplasts, although other ATP sources may also satisfy this ATP requirement.

Combining eqns (1.1, 1.6-1.7) and considering photorespiratory CO_2 release give the equation for gross CO_2 assimilation rate in terms of ATP ($A_{g,atp}$)

$$A_{\mathrm{g,atp}} = (1-0.5v_{\mathrm{o/c}})\frac{J_{\mathrm{atp}}}{d_{\mathrm{atp}}} = \frac{(1-0.5v_{\mathrm{o/c}})\frac{2+f_{\mathrm{Q}}-f_{\mathrm{CET}}(1-2f_{\mathrm{NDH}})}{h(1-f_{\mathrm{CET}}+r_{2/1})}\Phi_2 I_{\mathrm{abs}}}{3+3.5v_{\mathrm{o/c}}+1v_{\mathrm{n/c}}+c_{\mathrm{starch}}(1-0.5v_{\mathrm{o/c}}-v_{\mathrm{r/c}})+[2+a''-(n+1+a'')a](1+\phi)} \quad (1.8)$$

Quantum yield of CO_2 assimilation in terms of ATP ($\Phi_{CO2,atp}$) would be

$$\Phi_{CO2,atp} = \frac{A_{g,atp}}{I_{abs}} = \frac{(1-0.5v_{o/c})\frac{2+f_Q-f_{CET}(1-2f_{NDH})}{h(1-f_{CET}+r_{2/1})}\Phi_2}{3+3.5v_{o/c}+1v_{n/c}+c_{starch}(1-0.5v_{o/c}-v_{r/c})+[2+a''-(n+1+a'')a](1+\phi)} \quad (1.9)$$

Relationships between parameters f_{CET} and a

For the most efficient use of energy, neither NADPH nor ATP should be overproduced or under-utilised. A balance between NADPH and ATP in their production and utilisation is also metabolically important (Kramer & Evans 2011). To achieve that, $\Phi_{CO2,nadph}$ and $\Phi_{CO2,atp}$ should be equal, i.e.

$$\frac{(1-0.5v_{o/c})(1-f_{CET})\Phi_2}{(1-f_{CET}+r_{2/1})[l_e+2a(1+\phi)]} = \frac{(1-0.5v_{o/c})[2+f_Q-f_{CET}(1-2f_{NDH})]\Phi_2}{h(1-f_{CET}+r_{2/1})\{l_a+[2+a''-(n+1+a'')a](1+\phi)\}} \quad (1.10a)$$

where two lumped terms $l_e = 4 + 4v_{o/c} + 10v_{n/c}$ and $l_a = 3 + 3.5v_{o/c} + 1v_{n/c} + c_{starch}(1 - 0.5v_{o/c} - v_{r/c})$ are introduced to make eqn (1.10a) shorter. Simplifying gives

$$\frac{1-f_{CET}}{l_e+2a(1+\phi)} = \frac{2+f_Q-f_{CET}(1-2f_{NDH})}{h\{l_a+[2+a''-(n+1+a'')a](1+\phi)\}} \quad (1.10b)$$

Solving eqn (1.10b) for f_{CET} gives:

$$f_{CET} = 1 - \frac{[l_e+2a(1+\phi)](1+f_Q+2f_{NDH})}{h\{l_a+[2+a''-(n+1+a'')a](1+\phi)\}-[l_e+2a(1+\phi)](1-2f_{NDH})} \quad (1.11)$$

Eqn (1.11) suggests a hyperbolic relationship that f_{CET} decreases with increasing *a* (see the Figure at the end of Supporting Notes S1) if other parameters stay invariant (Table S2). This relationship means that a higher value of parameter *a* would generate more ATP via mitochondrial NADH oxidation and LET such that there is a lower requirement for CET to provide ATP in support of the C_4 cycle.

Estimating parameters f_{CET} and a from the measured Φ_{CO2}

Neither f_{CET} nor *a* is amenable to direct experimental measurement. We developed equations to estimate them based on values of Φ_{CO2}, which can easily be measured experimentally.

As discussed above, our model set that $\Phi_{CO2,nadph} = \Phi_{CO2,atp} = \Phi_{CO2}$. Eqn (1.5) can be reformulated as

$$\Phi_{CO2}[l_e + 2a(1+\phi)] = \frac{(1-0.5v_{o/c})(1-f_{CET})\Phi_2}{1-f_{CET}+r_{2/1}} \quad (1.12)$$

Substituting (1− f_{CET}) from eqn (1.11) into eqn (1.12) and arranging give

$$\Phi_{CO2} = \frac{(1-0.5v_{o/c})\Phi_2(1+f_Q+2f_{NDH})}{[l_e+2a(1+\phi)](1+f_Q+2f_{NDH})+r_{2/1}[hl_a-(1-2f_{NDH})l_e]+r_{2/1}\{h[2+a''-(n+1+a'')a]-2a(1-2f_{NDH})\}(1+\phi)} \quad (1.13)$$

Rearranging eqn (1.13) to solve for *a* gives

$$a = \frac{\frac{(1-0.5\nu_{o/c})\Phi_2(1+f_Q+2f_{NDH})}{\Phi_{CO2}} - l_e[1+f_Q+2f_{NDH}-r_{2/1}(1-2f_{NDH})] - [l_a+(2+a'')(1+\phi)]r_{2/1}h}{(1+\phi)\{2(1+f_Q+2f_{NDH})-r_{2/1}[h(n+1+a'')+2(1-2f_{NDH})]\}} \quad (1.14)$$

Once parameter *a* is solved, f_{CET} can be solved from eqn (1.11).

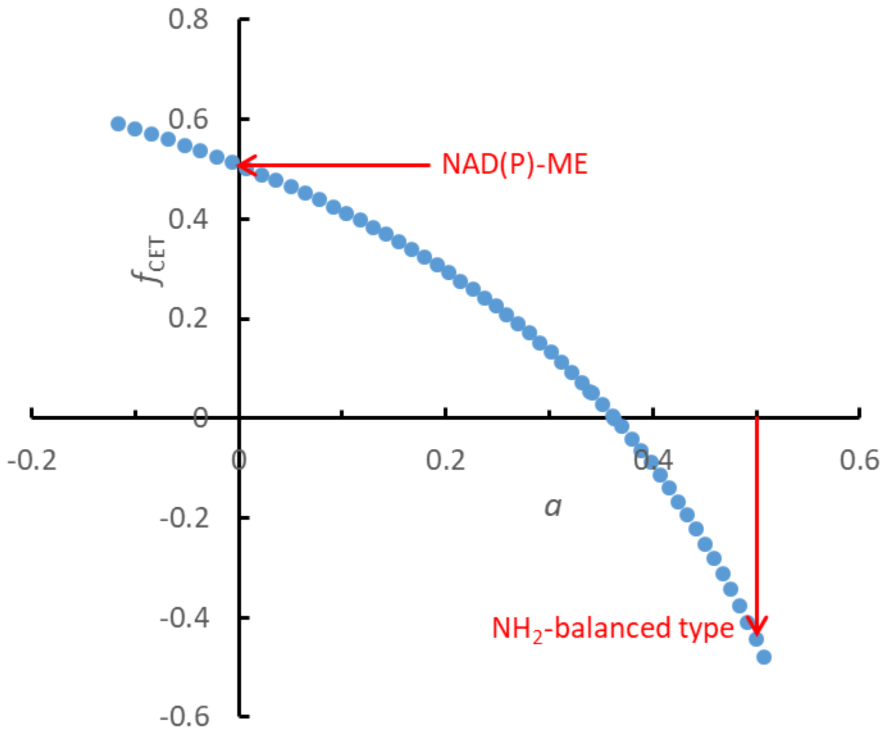

Figure Theoretical relationship between the required fraction of cyclic electron transport (f_{CET}) and parameter *a*, the fraction of oxaloacetate (OAA) that is reduced to malate in M cells for being shuttled to BS mitochondria where malate is decarboxylated by NAD-ME to release NADH that drives mitochondrial electron transport to generate ATP (here the ATP : NADH ratio *n* is assumed to be 3). The case where $a = 0$ and $f_{CET} = c.\ 0.5$ represents the classically-defined NADP-ME or NAD-ME subtypes. The case where $a = 0.5$ and $f_{CET} = -0.48$ represents the NH_2-flux balanced type discussed in the main text. Values where either *a* or $f_{CET} < 0$ are physiologically irrelevant, merely representing mathematical extrapolation of eqn (1.11). The type with only the NH_2-flux balance has the high *a* (0.5), meaning high mitochondrial electron transport generated ATP; and this, combined with high LET that also produces some ATP, yields ATP surplus that would need a mathematically negative CET for a physiologically balanced NADPH:ATP ratio.

Notes S2 Extending the model of Yin & Struik (2018) to accommodate the proposed mixed PEP-CK type

The model of Yin & Struik (2018) contains a simple module for light absorption by M and BS cells, and algorithms for both cell type-specific energy production and energy demand, and suits for three classically defined subtypes and other types. We now extend the model for algorithms to account for the proposed mixed PEP-CK type in Fig. 1.

Extended model for cell-type-specific NADPH and ATP production

The model for cell-type-specific NADPH and ATP production uses the following measurable traits as input: (1) leaf chlorophyll content (μmol m^{-2}), (2) fraction of chlorophyll in BS cells, (3) fraction of PSI in BS cells, and (4) fraction of PSII in BS cells. The main output of the model, among others, are fractions of NADPH and of ATP that are produced in BS or in M cells. The model was constructed on the basis that NADPH and ATP for the Calvin cycle come from LET while any shortfall of ATP for supporting the CCM cycle and other processes come from CET. This was modelled as eqn A1 in their Derivation A of Supporting Methods S1 of Yin & Struik (2018). The mixed PEP-CK type as proposed in Fig. 1 has a different NADPH:ATP ratio, compared with any classically-defined subtypes. To model this mixed type, two terms (*p* and *w*; see below or Table S1 for their definition) in that equation may need to be adjusted. However, as the total minimum requirement for NADPH per CO_2 assimilated in this mixed type is 2 + *a*, mathematically still the same as in the classically defined PEP-CK subtype, the formula for required LET must stay the same. It follows that ATP produced from LET (the term *p*) stays the same, and the final equation for the term *p* in the model of Yin & Struik (2018), eqn (17) in their Supporting Methods S1, still applies.

However, the term *w*, which defines the required ATP for the CCM cycle that has to come from CET, needs to be adjusted. The final equation of Yin & Struik (2018) for *w*, their eqn (21) in their Supporting Methods S1, can be rewritten as:

$$w = \varphi(1+\phi) + [3 - 4(\frac{2+f_Q}{h})](1+v_{o/c}) - ATP_{add} \quad (2.1)$$

where φ is ATP required for the CCM cycle; the term $(2+f_Q)/h$ is ATP produced per LET, so the middle term as a whole refers to the shortfall of ATP from LET in meeting the Calvin cycle and photorespiratory cycle if $h > 4$ and/or if $f_Q < 1$; ATP_{add} is ATP produced from the operation of other processes that consume electrons of LET but little ATP, so ATP produced from this portion of LET can be used to decrease the requirement for CET. ATP_{add} consists of:

$$ATP_{\mathrm{add}} = \frac{2+f_{\mathrm{Q}}}{h}[2a(1+\phi)] + IV \tag{2.2}$$

where the first part is the ATP produced from LET in M cells that provides NADPH to reduce OAA to malate, the second term *IV* is an intermediate variable standing for ATP saved from nitrate reduction, photorespiration and starch synthesis:

$$IV = 10\nu_{\mathrm{n/c}}(2+f_{\mathrm{Q}})/h - 1\nu_{\mathrm{n/c}} - 0.5\nu_{\mathrm{o/c}} - c_{\mathrm{starch}}\left(1 - 0.5\nu_{\mathrm{o/c}} - \nu_{\mathrm{r/c}}\right) \tag{2.3}$$

Now to accommodate the mixed type as described in Fig. 1, **two** modifications are needed. **First**, the term φ in eqn (2.1) needs to be expanded from 2a for the classically-defined PEP-CK subtype to:

$$\varphi = 2a + 2(1-a)a' + 3(1-a)a'' \tag{2.4}$$

where two terms are added to 2a to account for the ATP required for PEP regeneration by PPDK for NAD(P)-ME or PEP-CK(PK) and PEP-CK(PP) pathways, respectively (see Fig. 1). Applying the equation for the NH_2-flux balance in Fig. 1 to eqn (2.4) gives:

$$\varphi = 2 - 2a + (1-a)a'' \tag{2.4a}$$

Second, eqn (2.2) for ATP_{add} should become:

$$ATP_{\mathrm{add}} = [\frac{2+f_{\mathrm{Q}}}{h}(2a) + (na - a)](1+\phi) + IV \tag{2.5}$$

where ($na-a$) is the surplus of ATP from mitochondrial NADH oxidation that is not used for PEP-CK. This is necessary because, unlike the classically-defined PEP-CK subtype where ATP from the mitochondrial NADH oxidation just suffices to fuel PEP-CK, the mixed type has a surplus that would alleviate the requirement for CET.

Extended model for cell type-specific NADPH and ATP demand

Basic cell-type specific NADPH demands are $2(1-\gamma)+a(1+\phi)$ and 2γ for M and BS cells, respectively, for the classically defined PEP-CK subtype, where γ is the fraction of 3-PGA reduction that takes place in BS cells (Yin & Struik 2018). Now for the mixed PEP-CK type proposed in Fig. 1, there is a need to consider possible extra amount of NADPH required in M cells to reduce OAA to malate in case of some NADP-ME decarboxylation. We denote b as the fraction of the second decarboxylation category in Fig. 2 that is NADP-ME, then the extra amount of NADPH required in M cells is $(1-a)a'b(1+\phi)$ per CO_2 assimilation, where $(1-a)a'$ can also be expressed as $[1-2a-(1-a)a'']$ from the NH_2-flux balance. So the basic demand for NADPH in M cells is:

$$d_{\mathrm{nadph,M}} = 2(1-\gamma) + a(1+\phi) + [1 - 2a - (1-a)a'']b(1+\phi) \tag{2.6}$$

The NADPH released from malate decarboxylation by NADP-ME in BS chloroplasts could be used to reduce the demand for NADPH by 3-PGA reduction in BS cells. So the basic demand for NADPH in BS cells is:

$$d_{\mathrm{nadph,BS}} = 2\gamma - [1 - 2a - (1 - a)a'']b(1 + \phi) \quad (2.7)$$

For the classically defined PEP-CK subtype, basic ATP demand in M cells is $2a(1+\phi)+2(1-\gamma)$ (where $2a$ is the minimum ATP requirement by PPDK to regenerate PEP), and basic ATP demand in BS cells is $1+2\gamma$, where 1 is ATP required per RuBP regeneration that takes place in BS cells (Yin & Struik 2018). Now for the mixed PEP-CK type proposed in Fig. 1, there are two additional sources of ATP demands for PEP regeneration by PPDK for NAD(P)-ME or PEP-CK(PK) and PEP-CK(PP) pathways, respectively; so the total ATP demand by PPDK would be $[2a+2(1-a)a'+2(1-a)a''](1+\phi)$, which can be simplified to $(2-2a)(1+\phi)$ when applying the rule for NH_2-flux balance. This plus the ATP consumption by 3-PGA reduction would make the total ATP consumption in M cells per CO_2 assimilation as

$$d_{\mathrm{atp,M}} = 2(1 - \gamma) + (2 - 2a)(1 + \phi) \quad (2.8)$$

The ATP demand in BS cells of this mixed PEP-CK subtype should include (see Fig 1): (1) ATP required to fuel PEP-CK, which is $1(1-a)(1-a'-a'')(1+\phi)$, (2) ATP lost to entropy in the PEP phosphatase dependent pathway, which is $1(1-a)a''(1+\phi)$, and (3) ATP for RuBP regeneration and 3-PGA reduction, $1+2\gamma$. However, NADH-oxidation generates ATP, which is $na(1+\phi)$. So, taking these together and considering the rule for NH_2-flux balance give net ATP demand in BS cells in this mixed type:

$$d_{\mathrm{atp,BS}} = 1 + 2\gamma + [(1 - a)a'' - (n - 1)a](1 + \phi) \quad (2.9)$$

These cell-type-specific NADPH and ATP requirements for CCM and Calvin cycles, plus those required for photorespiratory cycle, nitrate reduction and starch synthesis, are summarised in Table S3.

Notes S3 Extending the C_4 submodel in crop model GECROS to accommodate the C_4 ideotype

The crop model GECROS (v4.0) was described by Yin & Struik (2017). Its submodel for C_4 leaf photosynthesis was a modified version of the model of von Caemmerer & Furbank (1999). The basic equations relevant to our analysis here are electron transport limited rate of PEP carboxylation (V_p) and gross rate of CO_2 assimilation (A_g):

$$V_p = xJ_2z/\varphi \quad (3.1)$$

where J_2 is total electron transport rate passing PSII and z is given in eqn (1.6), and

$$A_g = \frac{(C_c - \gamma_* O)x_1}{C_c + x_2 O}, \quad \text{with } x_1 = \left(1 - \frac{f_{pseudo}}{1 - f_{CET}}\right)\frac{J_2}{4} \text{ and } x_2 = 2\gamma_* \quad (3.2)$$

where C_c is the level of CO_2 at the carboxylating sites of Rubisco, O is the level of O_2 at the same sites, γ_* is the half of the inverse of Rubisco specificity for CO_2, and f_{pseudo} is the fraction of the total PSI electron flux for the basal pseudocyclic pathway (equivalent to accounting for the electron consumption by nitrate reduction as described in Supporting Notes S1 and S2). Eqn (3.2), the NADPH-limited form for CO_2 assimilation rate, was used because the equivalent ATP-limited form (as originally proposed for the C_4 model) where x_1 becomes $(1-x)J_2z/3$ and x_2 is $7\gamma_*/3$, would predict an increased rate of CO_2 assimilation with increasing f_{CET}, which is not physiologically logical (see Yin & Struik 2017).

To accommodate our C_4 ideotype, the following revisions have to be made here:

(1) Setting f_{CET} to 0.

(2) Changing the chloroplastic ATP requirement for the CCM cycle (φ) from 2 for malic-enzyme subtypes (von Caemmerer & Furbank 1999) to:

$$\varphi = 2 - (n+1)a \quad (3.3)$$

for the ideotype (where n is the ATP:NADH ratio, 3 or 2.5; and a = 0.36 or 0.4, see the main text).

(3) Changing the factor for ATP partitioned to the CCM cycle (x) from 0.4 for the NAD(P)-ME subtypes (von Caemmerer & Furbank 1999) to:

$$x = \frac{2-(n+1)a}{5-(n+1)a} \quad (3.4)$$

This gives that x is *c*. 0.16-0.17 for the ideotype (Table 1).

(4) Changing the stoichiometric coefficient in eqn 3.2 from 4 to (4 + 2a), i.e.:

$$x_1 = \left(1 - \frac{f_{pseudo}}{1 - f_{CET}}\right)\frac{J_2}{4+2a} \quad (3.5)$$

This accounts for the additional a mol NADPH required per mol CO_2 assimilated.

All other algorithms and parameter values were the same as described by Yin &Struik (2017).

Table S1 Definitions and units of model symbols

Symbol	Definition	Unit
a	Fraction of OAA that is reduced in M cells to malate moving to BS mitochondria for driving mitochondrial electron transport to produce ATP	–
a'	Fraction of remaining OAA that follow the NAD(P)-ME or PEP-CK(PK) in Fig. 1	–
a"	Fraction of remaining OAA that follow the PEP-CK(PP) pathway in Fig. 1	–
A_g	Gross rate of CO_2 assimilation	μmol m^{-2} s^{-1}
$A_{g,atp}$	ATP-determined gross rate of CO_2 assimilation	μmol m^{-2} s^{-1}
$A_{g,nadph}$	NADPH-determined gross rate of CO_2 assimilation	μmol m^{-2} s^{-1}
A_{max}	Light-saturated maximum net rate of leaf CO_2 assimilation	μmol m^{-2} s^{-1}
b	Fraction of the *a*' part that belongs to the NADP-ME type (see Fig. 1)	–
c_{starch}	ATP cost for starch synthesis	mol (mol C)$^{-1}$
d_{atp}	Demand for chloroplastic ATP per CO_2 assimilation	mol mol^{-1}
$d_{atp,BS}$	Demand in BS cells for chloroplastic ATP per CO_2 assimilation	mol mol^{-1}
$d_{atp,M}$	Demand in M cells for chloroplastic ATP per CO_2 assimilation	mol mol^{-1}
d_{nadph}	Demand for NADPH per CO_2 assimilation	mol mol^{-1}
$d_{nadph,BS}$	Demand in BS cells for NADPH per CO_2 assimilation	mol mol^{-1}
$d_{nadph,M}$	Demand in M cells for NADPH per CO_2 assimilation	mol mol^{-1}
f_{CET}	Fraction of the PSI electron flux that follow the cyclic electron transport (CET)	–
f_{NDH}	Fraction of CET that follow the NAD(P)H dehydrogenase-dependent pathway	–
f_Q	Fraction of electrons at plastoquinone that follow the Q-cycle	–
h	Protons required per ATP synthesis	mol mol^{-1}
I_{abs}	Irradiance absorbed by leaf photosynthetic pigments	μmol m^{-2} s^{-1}
J_{atp}	Rate of ATP production	μmol m^{-2} s^{-1}
J_{nadph}	Rate of NADPH production	μmol m^{-2} s^{-1}
l_a	Lumped term for ATP requirement per unit of CO_2 assimilated	mol mol^{-1}
l_e	Lumped term for electron requirement per unit of CO_2 assimilated	mol mol^{-1}
n	ATP produced per NADH oxidation	mol mol^{-1}
p	Required ATP that is from linear electron transport (LET)	mol mol^{-1}
$r_{2/1}$	PSII : PSI photochemical efficiency ratio	–
$v_{n/c}$	Nitrogen assimilation to RuBP carboxylation ratio	–
$v_{o/c}$	RuBP oxygenation to RuBP carboxylation ratio	–
$v_{r/c}$	Day respiration to RuBP carboxylation ratio	–
w	Required ATP for the C_4 cycle that is from cyclic electron transport (CET)	mol mol^{-1}
x	Proportion of the chloroplastic ATP that is used to support the C_4 cycle	–
z	Factor for ATP production per LET when CET runs simultaneously	mol mol^{-1}
α	Fraction of PSII that is in BS cells	–
γ	Fraction of 3-PGA reduction that takes place in BS cells	–
γ_{atp}	Fraction of ATP for 3-PGA reduction that is consumed in BS cells	–
γ_{nadph}	Fraction of NADPH for 3-PGA reduction that is consumed in BS cells	–
ϕ	Leakiness	–
Φ_2	Photochemical efficiency of PSII electron transport (under limiting light	mol mol^{-1}
Φ_{CO2}	Quantum yield for CO_2 assimilation (under limiting light conditions)	mol mol^{-1}
$\Phi_{CO2,atp}$	ATP-determined quantum yield for CO_2 assimilation	mol mol^{-1}
$\Phi_{CO2,nadph}$	NADPH-determined quantum yield for CO_2 assimilation	mol mol^{-1}
φ	Chloroplastic ATP required per C_4 cycle	mol mol^{-1}
ρ_2	Factor for excitation partitioning to PSII	–

Table S2 Indicative values of model input parameters used in the analysis

Symbol	Definition	Value	Source
a”	Fraction of the remaining OAA that follows the PEP-CK(PP) pathway as defined in Fig. 1	0	Most likely value (see the main text)
c_{starch}	ATP cost for starch synthesis	0.167 mol ATP mol^{-1} carbon	Noctor & Foyer (1998)
f_{NDH}	Fraction of cyclic electron transport that follows the NAD(P)H dehydrogenase-dependent pathway	0	Assumed value (Yin & Struik 2012)
f_Q	Fraction of electrons at plastoquinone that follow the Q-cycle	1	Furbank et al. (1990); Yin & Struik (2012)
h	Protons required per ATP synthesis	4	Steigmiller et al. 2008; Yin & Struik 2012
n	ATP produced per NADH oxidation	3, or 2.5	Ferguson (1986), or Hinkle et al. (1991)
$r_{2/1}$	PSII : PSI photochemical efficiency ratio	0.85	Genty & Harbinson (1996)
$v_{n/c}$	Nitrogen assimilation to RuBP carboxylation ratio	0.0286	Kanai & Edwards (1999)
$v_{o/c}$	RuBP oxygenation to RuBP carboxylation ratio	0.05	A common value for C_4 photosynthesis
$v_{r/c}$	Day respiration to RuBP carboxylation ratio	0.025	Data of Yin et al. (2011)
ϕ	Leakiness	0.16	Yin & Struik (2012)
Φ_2	Photochemical efficiency of PSII electron transport under limiting light conditions	0.8 mol mol^{-1}	Genty & Harbinson (1996)

New Phytologist

Table S3 Formulae for calculating cell-type-specific NADPH and ATP demands per CO_2 assimilation in the mixed PEP-CK type proposed in Fig. 1

	NADPH	ATP
M cells	$2(1-\gamma)+\{a+[1-2a-(1-a)a'']b\}(1+\phi)+x_1$	$2(1-\gamma)+(2-2a)(1+\phi)+x_3$
BS cells	$2\gamma-[1-2a-(1-a)a'']b(1+\phi)+x_2$	$1+2\gamma+[(1-a)a''-(n-1)a](1+\phi)+x_4$
Total	$2+a(1+\phi)+x_1+x_2$	$3+[2+(1-a)a''-(n+1)a](1+\phi)+x_3+x_4$

$x_1=1.5v_{o/c}(1-\gamma)+5v_{n/c}$, $x_2=1.5v_{o/c}\gamma+0.5v_{o/c}$, $x_3=1.5v_{o/c}(1-\gamma)+v_{n/c}$ and $x_4=1.5v_{o/c}\gamma+2v_{o/c}+0.167(1-0.5v_{o/c}-v_{r/c})$, where $v_{o/c}$, $v_{n/c}$ and $v_{r/c}$ are as defined earlier, referring to the ratios of oxygenation, nitrate reduction and day respiration to carboxylation, respectively. It is assumed in these formulae that (i) in the photorespiratory cycle, only NADPH and ATP consumption during the 3-PGA reduction phase (i.e. $1.5v_{o/c}$ NADPH and $1.5v_{o/c}$ ATP, von Caemmerer 2000) is partitioned between BS and M cells, whereas the remaining $0.5v_{o/c}$ NADPH and $2v_{o/c}$ ATP consumption by the photorespiratory cycle takes place in BS cells; and (ii) nitrate reduction predominantly takes place in the M cells whereas starch synthesis predominantly takes place in the BS cells (Furbank et al. 1985; Kanai & Edwards 1999; Majeran et al. 2008).

References

von Caemmerer S. 2000. *Biochemical models of leaf photosynthesis*. Clayton, VIC, Collingwood, Australia: CSIRO Publishing.

von Caemmerer S, Furbank RT. 1999. Modeling C_4 photosynthesis. In: Sage RF, Monson RK, eds. *C_4 Plant Biology*. Toronto, Canada: Academic Press, 173-211.

Farquhar GD, von Caemmerer S, Berry JA. 1980. A biochemical model of photosynthetic CO_2 assimilation in leaves of C_3 species. *Planta* **149:** 78-90.

Ferguson SJ. 1986. The ups and downs of P/O ratios (and the question of non-integral coupling stoichiometries for oxidative phosphorylation and related processes). *Trends in Biochemical Sciences* **11:** 351-353.

Furbank RT, Jenkins CLD, Hatch MD. 1990. C_4 photosynthesis: Quantum requirement, C_4 acid overcycling and Q-cycle involvement. *Australian Journal of Plant Physiology* **17:** 1-7.

Furbank RT, Stitt M, Foyer CH. 1985. Intercellular compartmentation of sucrose synthesis in leaves of *Zea mays* L. *Planta* **164:** 172-178.

Genty B, Harbinson J. 1996. Regulation of light utilization for photosynthetic electron transport. In: Baker NR, ed. *Photosynthesis and the Environment.* Vol 5 book series 'Advances in Photosynthesis and Respiration'. Dordrecht, The Netherlands: Kluwer Academic Publishers, 67-99.

Hinkle PC, Kumar MA, Resetar A, Harris DL. 1991. Mechanistic stoichiometry of mitochondrial oxidative phosphorylation. *Biochemistry* **30:** 3576-3582.

Ishikawa N, Takabayashi A, Sato F, Endo T. 2016. Accumulation of the components of cyclic electron flow around photosystem I in C_4 plants, with respect to the requirements for ATP. *Photosynthesis Research* **129:** 261-277.

Kanai R, Edwards GE. 1999. The biochemistry of the C_4 photosynthesis. In: Sage RF, Monson RK, eds. *C_4 Plant Biology*. Toronto, Canada: Academic Press, 49-87.

Kramer DM, Evans JR. 2011. The importance of energy balance in improving photosynthetic productivity. *Plant Physiology* **155:** 70-78.

Majeran W, Zybailov B, Ytterberg AJ, Dunsmore J, Sun Q, van Wijk KJ. 2008. Consequences of C_4 differentiation for chloroplast membrane proteomes in maize mesophyll and bundle sheath cells. *Molecular and Cellular Proteomics* **7:** 1609-1637.

Noctor G, Foyer CH. 1998. A re-evaluation of the ATP:NADPH budget during C_3 photosynthesis: a contribution from nitrate assimilation and its associated respiratory activity? *Journal of Experimental Botany* **49:** 1895-1908.

Petersen J. Förster K, Turina P, Gräber P. 2012. Comparison of the H^+/ATP ratios of the H^+-ATPsynthases from yeast and from chloroplast. *Proceedings of the National Academy of Sciences, USA* **109:** 11150-11155.

Seelert H, Poetsch A, Dencher NA, Engel A, Stahlberg H, Müller DJ. 2000. Proton powered turbine of a plant motor. *Nature* **405:** 418-419.

Steigmiller S, Turina P, Gräber P. 2008. The thermodynamic H^+/ATP ratios of the H^+-ATPsynthases from the chloroplasts and *Escherichia coli*. *Proceedings of the National Academy of Sciences, USA* **105:** 3745-3750.

Yin X, Struik PC. 2012. Mathematical review of the energy transduction stoichiometries of C_4 leaf photosynthesis under limiting light. *Plant, Cell & Environment* **35:** 1299-1312.

Yin X, Struik PC. 2017. Can increased leaf photosynthesis be converted into higher crop mass production? A simulation study for rice using the crop model GECROS. *Journal of Experimental Botany* **68:** 2345-2360.

Yin X, Struik PC. 2018. The energy budget in C_4 photosynthesis: insights from a cell-type-specific electron transport model. *New Phytologist* **218:** 986-998.

Yin X, Sun Z, Struik PC, van der Putten PEL, van Ieperen W, Harbinson J. 2011. Using a biochemical C_4-photosynthesis model and combined gas exchange and chlorophyll fluorescence measurements to estimate bundle-sheath conductance of maize leaves differing in age and nitrogen content. *Plant, Cell & Environment* **34:** 2183-2199.

Yin X, van Oijen M, Schapendonk AHCM. 2004. Extension of a biochemical model for the generalized stoichiometry of electron transport limited C_3 photosynthesis. *Plant, Cell & Environment* **27:** 1211-1222.
